# Integrating the Hallmarks of Aging Throughout the Tree of Life: A Focus on Mitochondrial Dysfunction

**DOI:** 10.3389/fcell.2020.594416

**Published:** 2020-11-26

**Authors:** Sanne van der Rijt, Marte Molenaars, Rebecca L. McIntyre, Georges E. Janssens, Riekelt H. Houtkooper

**Affiliations:** Laboratory Genetic Metabolic Diseases, Amsterdam Gastroenterology Endocrinology Metabolism, Amsterdam Cardiovascular Sciences, Amsterdam UMC, University of Amsterdam, Amsterdam, Netherlands

**Keywords:** aging, mitochondria, interplay, hallmarks of aging, tree of life

## Abstract

Since the identification and definition of the hallmarks of aging, these aspects of molecular and cellular decline have been most often described as isolated or distinct mechanisms. However, there is significant evidence demonstrating interplay between most of these hallmarks and that they have the capacity to influence and regulate one another. These interactions are demonstrable across the tree of life, yet not all aspects are conserved. Here, we describe an integrative view on the hallmarks of aging by using the hallmark “mitochondrial dysfunction” as a focus point, and illustrate its capacity to both influence and be influenced by the other hallmarks of aging. We discuss the effects of mitochondrial pathways involved in aging, such as oxidative phosphorylation, mitochondrial dynamics, mitochondrial protein synthesis, mitophagy, reactive oxygen species and mitochondrial DNA damage in relation to each of the primary, antagonistic and integrative hallmarks. We discuss the similarities and differences in these interactions throughout the tree of life, and speculate how speciation may play a role in the variation in these mechanisms. We propose that the hallmarks are critically intertwined, and that mapping the full extent of these interactions would be of significant benefit to the aging research community.

## Introduction

### The Nine Hallmarks of Aging

Aging is a multifactorial and complex process, affecting organisms at the molecular, cellular, tissue, and system levels (Gems and Partridge, 2013; Cohen, 2018). This progressive deterioration throughout life leads to age-related diseases such as cardiovascular diseases, cancer, osteoarthritis, diabetes mellitus type II and neurodegenerative diseases such as Alzheimer’s Disease (AD) and Parkinson’s Diseases (PD) (López-Otín et al., 2013; Franceschi et al., 2018). While lifespan has steadily increased in humans (Oeppen and Vaupel, 2002), many of these diseases remain prevalent and possess no cures. With the aim of uncovering mechanisms of aging and discovering interventions that promote healthier aging, scientists have utilized model organisms from across the phylogenetic tree. The most common model organisms are short lived, with well annotated genomes and molecular toolkits to intervene genetically in aging, such as *S. cerevisiae*, *C. elegans*, *D. melanogaster*, and *M. musculus* (Mitchell et al., 2015). Outside these traditional model organisms, aging researchers have studied extremely long-lived species, such as Hydra and the naked mole rat (Buffenstein, 2005; Boehm et al., 2012), as well as emerging models for aging research such as the killifish (Harel and Brunet, 2016).

Geroprotective interventions, either environmental or genetic, can extend the lifespan of model organisms (Mitchell et al., 2015). Through such experiments, nine hallmarks of aging have been defined, with indications that these are also conserved in humans (López-Otín et al., 2013). While more hallmarks are likely to be described in the future, as was the case for the hallmarks of cancer (Hanahan and Weinberg, 2011), we provide evidence of the interplay of between these current nine hallmarks of aging. The nine cellular and molecular hallmarks of aging are divided into three groups; (a) the primary hallmarks, (b) antagonistic hallmarks, and (c) integrative hallmarks (López-Otín et al., 2013). The primary hallmarks, which are unequivocally deleterious to the cell, include genomic instability, telomere attrition, epigenetic alterations, and loss of proteostasis. The antagonistic hallmarks, which are beneficial at low levels but at high levels become deleterious, are deregulated nutrient sensing, cellular senescence, and mitochondrial dysfunction. Finally, the integrative hallmarks, affecting tissue homeostasis and function, are stem cell exhaustion and altered intercellular communication. These hallmarks of aging have been presented throughout various research disciplines as nine separate hallmarks, and, while possessing crosstalk, are still considered largely independent. In this review, we provide evidence of the interplay between hallmarks, by highlighting one, mitochondrial dysfunction, and how it interacts with all others. In doing so, we aim to provide a broader view of the mitochondria’s role in the aging process throughout the tree of life and suggest reflection on the integrative nature of complex biological processes such as aging.

### Mitochondrial Dysfunction and Aging

The role of mitochondrial dysfunction in aging is well documented and for comprehensive reviews on mitochondrial function in aging we refer the reader to Sun et al. (2016), Zhang et al. (2018), Bar-Ziv et al. (2020), Rath (2020). Here we summarize the various aspects of mitochondrial biology and aging across the prominent model organisms that are relevant to this review and the hallmark of mitochondrial dysfunction.

Mitochondria generate energy in the cell, via the electron transport chain (ETC) and oxidative phosphorylation (OXPHOS). In general, mitochondrial function is impaired with age, marked by altered mitochondrial respiration, decreased energy production in the form of adenosine triphosphate (ATP), as well as widespread changes in metabolites associated with mitochondrial function (Lesnefsky and Hoppel, 2006; Houtkooper et al., 2011). Moreover, as a consequence of energy production, mitochondria also produce reactive oxygen species (ROS), which are formed during OXPHOS, causing oxidative damage to the mitochondria (Lesnefsky and Hoppel, 2006). ROS can contribute to damage to mitochondrial DNA (mtDNA), oxidation of mitochondrial proteins, less effective electron transport chain and impaired quality control in mitophagy (Bratic and Larsson, 2013; López-Otín et al., 2013). Yet, antioxidants do not provide lifespan benefits in model organisms through ROS scavenging, suggesting that ROS generation and oxidative stress do not themselves cause aging (Schulz et al., 2007). Instead, ROS have even been described as important signaling molecules in the cell, eliciting protective gene expression beneficial to longevity (Schulz et al., 2007). In line with this, a systematic review showed that there was no beneficial effect of antioxidant supplementation on the mortality in patients with various diseases (Bjelakovic et al., 2012). Recently, comparative analysis between mammalian species ranging in longevity identified a correlation between low levels of specific OXPHOS complex I proteins, ROS production, and lifespan in a species-specific manner (Mota-Martorell et al., 2020). Therefore, speciation may affect the role of ROS in aging and vice versa. In other words, ROS might have evolved different roles and physiologically normal levels depending on the environment of the species. These different selective pressures result in differences in the role of ROS.

While mitochondrial dysfunction shows detrimental effects at old age, it can also provide protection via hormetic stresses that promote longevity, especially when targeted early in life. For instance, reduction of nuclear encoded OXPHOS proteins via RNAi leads to extended life span in when the treatment is initiated in the larval stages of *C. elegans* (Dillin et al., 2002; Houtkooper et al., 2013). The proteins of the OXPHOS complexes are encoded by both nuclear DNA (nDNA) and mtDNA. Inhibiting mitochondrial translation and thus lowering synthesis of mtDNA-encoded OXPHOS proteins extends lifespan in *C. elegans*, and the link between mitochondrial translation and lifespan is conserved in natural populations of mice (Houtkooper et al., 2013). Inhibiting mitochondrial translation causes a mitonuclear protein imbalance that activates the protective mitochondrial unfolded protein response (UPR^*mt*^). The UPR^*mt*^ is a stress response and one example of how dysfunctional mitochondria communicate to the nucleus where it activates mitochondrial chaperones and proteases, ROS detoxification enzymes, and mitochondrial protein import components (Nargund et al., 2015). In addition to UPR^*mt*^, several other pathways exist to protect mitochondria from intrinsic or extrinsic damage, including mitoCPR, MAGIC, UPR^*am*^, and mito-cytosolic translational balance (Molenaars et al., 2020a,b).

It is not only mitochondrial function that plays a role in aging, but also mitochondrial form. Mitochondria are constantly dynamically rearranging and degrading via the processes of fission, fusion and mitophagy. Mitochondria use these processes as quality control systems, degrading poorly-functional mitochondria on the go, and while specific proteins vary between model organisms, these processes are highly conserved (Liu et al., 2020a). Mitophagy involves the elimination of non-functional mitochondria, for instance upon their loss of membrane potential. Mitophagy is reduced throughout the aging process, and conversely, inducing mitophagy through genetic or pharmacological means leads to a longer lifespan (Wu et al., 2013; Ryu et al., 2016). Aging and age-related diseases are often accompanied by a lack of mitochondrial fusion, which leads to fragmented mitochondria (Chen et al., 2007; Houtkooper et al., 2013; Sharma et al., 2019), and altering mitochondrial dynamics of fission and fusion can extend lifespan in worms (Weir et al., 2017; Liu et al., 2020b).

Each of these aspects of mitochondrial dysfunction plays a significant role in aging. We next look beyond mitochondrial dysfunction as an individual hallmark and explore how these mitochondrial processes and age-related deteriorations interrelate and communicate with the other hallmarks of aging.

## Integrating Mitochondrial Function With the Primary Hallmarks of Aging

The primary hallmarks of aging are defined as unequivocally deleterious to the cell. This means that proper functioning of these processes is important for the viability of the cell and the dysfunction that occurs with age leads to cellular damage. Mitochondrial dysfunction interacts with each of these primary hallmarks, thus leading to progression of the aging process.

### Genomic Instability and Mitochondrial Dysfunction

During aging, DNA damage accumulates, for instance due to ROS or environmental sources such as radiation (Fakouri et al., 2019). When DNA damage occurs, the DNA damage repair (DDR) is triggered (Niedernhofer et al., 2018; Fakouri et al., 2019). DDR activates several pathways, such as apoptosis, repair, or senescence. Every day, over 10^4^ mutations occur in the genome (Niedernhofer et al., 2018). These mutations can be repaired by several mechanisms such as base excision repair, nucleotide excision repair, interstrand crosslink repair, non-homologous end joining and homologous recombination (Niedernhofer et al., 2018). The capacity for DNA repair decreases with aging, which results in accumulations of mutations with age and genomic instability (Niedernhofer et al., 2018).

The increasing amounts of DNA damage occurring during aging lead to activation of poly (ADP-ribose) polymerase 1 (PARP1), which serves as a DNA damage sensor and activator of various target proteins involved in the DNA damage response (Gupte et al., 2017). PARP1 uses nicotinamide adenine dinucleotide (NAD^+^) as a substrate in this process, and increased amounts of DNA damage therefore require large amounts of NAD^+^, resulting in a depletion of the NAD^+^ pool (Berger, 1985). This depletion of the NAD^+^ pool contributes to mitochondrial dysfunction considering that NAD^+^ acts as a co-factor to activate sirtuins. Sirtuins play important roles for mitochondrial function and longevity, including the activation the transcription factor peroxisome proliferator-activated receptor gamma, coactivator 1 alpha (PGC-1α), which stimulates mitochondrial biogenesis and function, and NAD^+^ depletion reduces this activation (Houtkooper et al., 2012). In line with this observation, preserving NAD^+^ by inhibiting PARP activity leads to healthier cells. Possible detrimental effects. Indeed, mice with mutations in PARP1 or PARP2 or those treated with pharmacological PARP inhibitors showed improved mitochondrial function and organismal fitness (Bai et al., 2011; Pirinen et al., 2014), and worms with PARP inhibition had extended lifespan (Mouchiroud et al., 2013). However, it should also be taken into account that PARP is important for DNA repair and PARP inhibition can possibly lead to genomic instability and cancer. Therefore, it should be investigated what are the long-term side effects of PARP inhibition and how these can be prevented (Bai et al., 2011).

Another avenue in which mitochondrial dysfunction and genomic instability directly meet is through the mutation of mtDNA genes. Mutations in mtDNA is generally 10-fold higher than in nuclear DNA (Wang et al., 2005). mtDNA damage can have various downstream consequences related to aging, such as damage in neurons, increasing the risk on neurodegenerative diseases such as AD (Wang et al., 2005). Another way by which mitochondria might contribute to DNA maintenance is through their role in nucleotide synthesis. Indeed, mitochondrial dysfunction leads to impaired synthesis of nucleotides (Tufi et al., 2014), and although not formally demonstrated one might expect downstream consequences on telomere attrition and genome stability. To summarize, genome instability, for instance caused by endogenous or exogenous DNA damage sources can damage mtDNA or indirectly impair mitochondrial function through NAD+.

### Telomere Attrition and Mitochondrial Dysfunction

All species must maintain the integrity of the ends of their chromosomes. How this is achieved varies greatly per species, for instance through (a) retrotransposons in *Drosophila melanogaster*, (b) having circular DNA and therefore no chromosome ends, such as with bacteria, or (c) possessing “telomeres” at the ends of chromosomes, such as in humans. When cells divide, telomeres become shorter, and when telomeres become too short, cells enter the state of senescence, which is an irreversible growth arrest. Cellular senescence is itself a hallmark of aging that contributes to age-related dysfunction and disease (López-Otín et al., 2013). Rapid telomere shortening can therefore coincide with an acceleration of aging and, indeed, studies in humans demonstrated that individuals with shorter telomeres have mortality rates twice as high as those with longer telomeres. Additionally, individuals with shorter telomeres have a significantly higher chance of having heart disease and age-related diseases compared to individuals with longer telomeres (Cawthon et al., 2003).

Mitochondria can directly contribute to an acceleration of telomere shorting, due to their production of ROS, which lead to DNA damage and telomere shortening (Liu et al., 2002). For example, cells treated with the protonophore carbonyl p-trifluoromethoxyphenylhydrazone (FCCP), a compound which depolarize the mitochondrial membrane potential and disrupts ATP synthesis, have increased ROS production (Liu et al., 2002) as well as shorter telomeres compared to untreated cells. Both ROS levels and shorter telomeres were rescued by treating the cells with ROS scavenger molecules, indicating that ROS leads to the shortening of telomeres (Liu et al., 2002). Similarly, irradiating cells with UV-A caused an increased level of 8-oxo-7,8-dihydro-2′-deoxyguanosine (8-oxodG), a marker of DNA oxidation, as well as accelerated telomere shortening (Kawanishi and Oikawa, 2004).

The presence of critically short telomeres leads to the activation of the tumor suppressor gene p53 in mice (Sahin et al., 2011). P53 itself is known to inhibit PGC-1α/β, leading to the inhibition of mitochondrial biogenesis, OXPHOS, decreased mitochondrial mass, less ATP production and increased ROS production (Sahin et al., 2011). Therefore, initial damage to telomeres by mitochondrial ROS can cause a cycle of increasing damage, whereby PGC-1α inhibition further impairs mitochondrial function, creating additional ROS, which then contributes to further telomere damage. This is in line with the observations of telomere shortening, oxidative stress and aging in PGC-1α knock-out mice (Xiong et al., 2015). Generally, mitochondrial dysfunction can cause telomere attrition, which inhibits PGC-1α/β, resulting in further mitochondrial dysfunction. Moreover, future work might reveal more crosstalk links.

### Epigenetics and Mitochondrial Dysfunction

Reversible modifications of DNA and chromosomes, called epigenetics, are another hallmark of aging (López-Otín et al., 2013). Out of all the epigenetic changes that occur, most is known regarding DNA methylation and histone modifications. DNA methylation occurs through the activity of DNA methyltransferases (DNMTs), on regions of cytosines followed by guanines (termed CpG islands). During aging, hypermethylation of these CpG regions occurs, while hypomethylation occurs outside of these CpG regions (Christensen et al., 2009). Histone modifications are also affected during aging. Trimethylation on the lysine 4 residue of histone 3 (H3K4me3) leads to transcriptional activation, while trimethylation of lysine 27 of histone 3 (H3K27me3) is a repressive mark. Knockdown of one of the complexes of the H3K4me3 methyltransferase results in a longer lifespan in *C. elegans* (Greer et al., 2010). Another epigenetic modification is through histone deacetylases (HDACs), removing acetyl groups on histones. HDACs regulate the chromatin structure and play a critical role in transcription regulation. HDAC inhibitor compounds and their effects on each of the hallmarks of aging have previously been reviewed (McIntyre et al., 2019). HDAC inhibitors can increase lifespan in yeast, worms and flies (Lee et al., 2012; McIntyre et al., 2019).

Mitochondrial dysfunction plays a role in determining epigenetics changes during aging. Increased ROS leads to changes in the methylome, inducing aspects of the epigenetic aging process (Kietzmann et al., 2017). ROS causes DNA lesions and the most common base lesion is 8-hydroxyguanine (8-OHdG), which is in fact used as a measure for the level of oxidative stress (Turk et al., 1995). 8-OHdG lesions in cells caused by ROS inhibit DNA methylation, in line with the hypothesis that mitochondrial dysfunction and ROS production may be a driver of altered DNA methylation observed during aging (Turk et al., 1995).

HDAC inhibitors such as suberoylanilide hydroxamic acid (SAHA) and trapoxin A result in mitochondrial elongation, leading to healthy aging (Lee et al., 2012). In *C. elegans*, mitochondrial stress can lead to changes in chromatin structures such as H3K9 di-methylation (Tian et al., 2016). Normally these marks lead to silenced chromatin, however, some parts open up which lead to the expression of the mitochondrial unfolded protein response (UPR^*mt*^), including activation of chaperones and quality control proteases (Tian et al., 2016). H3K27 demethylases, *jmjd-1.2* and *jmjd-1.3*, are activated when there is a perturbation in the ETC, which leads to the demethylation of H3K27 and UPR^*mt*^ activation, inducing longevity in *C. elegans* (Merkwirth et al., 2016).

Another study looked at a genome wide blood methylome profile, or DNA methylation age clock, in 656 individuals with the age between 19 and 101. Three methylation sites that change with age were found (Hannum et al., 2013). For instance, a missense mutation was found in the locus of GTP binding protein 10 (*GTPBP10*), located close to the gene *STEAP2*. The methylation state of *GTPBP10* also lowers the gene expression of *STEAP2* and influences the synthesis of iron and copper (Hannum et al., 2013), elements that are required for proper OXPHOS (Ohgami et al., 2006).

CpG methylation of 12 other genes were later associated with aging, and two of these genes encode proteins that affect mitochondrial function (D’Aquila et al., 2019). The genes ras-related protein 32 *(RAB32)* and mitochondrial rho GTPase 2 *(RHOT2)*, were hyper- and hypomethylated with age, respectively. Furthermore, *RAB32* mRNA expression was decreased with age and *RHOT2* expression was increased. *RAB32* encodes for a GTPase and is important for mitochondrial fission and fusion, mitophagy and apoptosis (Alto et al., 2002). *RHOT2* encodes for a protein in the outer mitochondrial membrane (OMM) and is important for the transport of molecules into the intermembrane space in the mitochondria, and also for mitochondrial fission and fusion (Fransson et al., 2006). These provide direct examples of how methylation state may influence mitochondrial biology during aging (D’Aquila et al., 2019).

To summarize, changes in mitochondrial biology with aging may result in increased ROS, which concomitantly alter epigenetic state at the DNA methylation level. Furthermore, DNA methylation as well as histone acetylation change during aging and impart alterations in gene expression of mitochondrial genes, creating a feedback loop of declining mitochondrial function.

### Impaired Proteostasis and Mitochondrial Dysfunction

Mitochondrial proteostasis has been extensively linked to aging (Jensen and Jasper, 2014; Moehle et al., 2019). Several pathways of mitochondrial proteostasis exist that restore mitochondrial function, including UPR^*am*^, which is activated by mistargeted mitochondrial precursor proteins, and mitoCPR which is activated upon mitochondrial import stress and (Wrobel et al., 2015; Weidberg and Amon, 2018). While these proteostasis pathways were predominantly described in yeast, the most extensively described pathway, UPR^*mt*^, was implicated in lifespan extension in worms, flies, and mice, suggesting a conserved role in the long-term maintenance of cellular homeostasis (Jovaisaite et al., 2014).

While the UPR^*mt*^ ensures proteostasis specifically for mitochondrial proteins as a relatively independent protein quality control system, cytosolic proteins are simultaneously synthesized, folded, maintained and degraded in the cytosol. Stress can cause the unfolding and aggregation of proteins, and cells have different mechanisms to maintain proteostasis: specifically molecular chaperones, stress-response transcription factors and protein degradation via autophagy (Hartl et al., 2011). Another study showed that in *C. elegans* the mitochondrial ETC is a central regulator of the age-related decline of cytosolic proteostasis (Labbadia et al., 2017). Many more of these cytosolic proteostasis mechanisms linked to longevity are highly interconnected with mitochondria (reviewed in D’Amico et al., 2017; Molenaars et al., 2020a).

For instance, slowing down the synthesis of new proteins, by knockdown of cytosolic ribosomal proteins, extends lifespan in the model organism *C. elegans* (Hansen et al., 2007; Pan et al., 2007). Linking this to mitochondria, slowing down cytosolic protein synthesis is one of the consequences of mitochondrial disturbances that extend lifespan in *C. elegans*. For instance, ROS generated from dysfunctional mitochondria activates GCN-2-dependent eIF2α phosphorylation, leading to reduced cytosolic protein synthesis (Baker et al., 2012). Moreover, when looking at translational efficiencies in *C. elegans* with dysfunctional mitochondria, mRNAs coding for elements of the translation machinery were decreased and those coding for the OXPHOS and autophagy pathways were increased (Molenaars et al., 2018). Additionally, in *C. elegans*, mitochondrial translation and dynamics can synergistically regulate lifespan. This lifespan extension was dependent on the autophagy and lysosome biogenesis regulator, *hlh-30* (*TFEB* in mammals) (Liu et al., 2020b), demonstrating a clear connection and communication between mitochondrial form and function with global cellular proteostasis.

Mitochondria are not only linked to cytosolic protein synthesis; accumulating evidence has shown that cytosolic proteins and aggregation-prone misfolded proteins can be translocated into the mitochondria. For instance, in yeast, cytosolic proteins prone to aggregation are imported into mitochondria for degradation which can be degraded via mitophagy (Ruan et al., 2017; Eldeeb and Fahlman, 2018).

In conclusion, both mitochondrial and cytosolic proteostasis, which can be linked via mito-cytosolic translational balance (Suhm et al., 2018; Molenaars et al., 2020b), are important for healthy aging. There is significant crosstalk between mitochondria and various aspects of proteostasis, both mitochondrial and cytosolic. At the same time there is a lot more to be discovered, especially with regard to the type of proteostatic response that is triggered depending on the context of the cellular or mitochondrial stress.

## Integrating Mitochondrial Function With the Antagonistic Hallmarks of Aging

The antagonistic hallmarks of aging are hallmarks that can have beneficial or deleterious effects on the cell, depending on the level of intensity (López-Otín et al., 2013). When regulated properly, these hallmarks are beneficial or protective, but can be deleterious when levels are too high, or unregulated.

### Deregulated Nutrient Sensing and Mitochondrial Dysfunction

Nutrient sensing pathways involve the detection and cellular adaptation to nutritional challenges, either scarcity or excess. The activity of nutrient sensing pathways changes with age. Some of the best described nutrient sensing pathways involved in aging are the insulin/IGF1 pathway, the mechanistic target of rapamycin (mTOR), the sirtuin pathway, and AMP-activated protein kinase (AMPK) pathway (Houtkooper et al., 2010). Insulin/IGF1 and mTOR are typically activated under conditions of nutrient excess, leading to the activation of anabolic responses including glucose uptake, protein synthesis and inhibition of autophagy. Inhibition of these pathways, for instance inhibition of mTOR complex 1 (mTORC1) with rapamycin leads to marked lifespan extension in model organisms, including mice (Harrison et al., 2009). AMPK and sirtuins are activated upon caloric restriction and lead to activation of various catabolic pathways as well as stress defense systems. Activation of AMPK and sirtuins leads to enhanced autophagy, mitochondrial biogenesis and stress response, culminating in prolonged lifespan (Pearson et al., 2008). Importantly, the anabolic and catabolic nutrient sensing pathways function in intricate networks with bidirectional molecular communication (Houtkooper et al., 2010).

It is evident that mitochondrial (dys)function and nutrient sensing are connected. Through translational and transcriptional mechanisms, mTOR can regulate mitochondrial biogenesis, functions and dynamics (Cunningham et al., 2007; Morita et al., 2013, 2017; Gandin et al., 2016). Translation of nDNA-encoded proteins of OXPHOS complex I and V and fission protein mitochondrial fission process 1 (MTFP1) are stimulated by mTORC1 (Morita et al., 2013, 2017). When mTORC1 and thus MTFP1 is inhibited, this will lead to fusion of mitochondria and survival of the cell. In addition, mTORC1 can stimulate transcription of mitochondrial genes of the ETC leading to sustain high ATP production in the cell (Morita et al., 2013). Another mechanism connecting mTOR with mitochondrial function involves mitophagy. Mitophagy is reduced during aging. In TSC2-null cells, which have high mTORC1 activity, the activity of mitophagy was reduced (Bartolomé et al., 2017). This resulted an accumulation of defective mitochondria, leading to a shorter lifespan (Palikaras et al., 2015; Bartolomé et al., 2017).

Mitochondrial biogenesis regulates intracellular energy metabolism, in response to decreased energy. One of the regulators of mitochondrial biogenesis is AMPK. Activation of AMPK leads to initiation of mitophagy, for instance by activating the autophagy activating kinase 1 (Ulk1) (Egan et al., 2011; Laker et al., 2017). Furthermore, AMPK is important in fission of mitochondria (Toyama et al., 2016). The induction of AMPK can increase lifespan of model organisms such as *C. elegans* by maintaining mitochondrial homeostasis through the regulation of mitochondrial dynamics (Weir et al., 2017). Besides this, excess in nutrients, for instance in glucose or amino acids, can also lead to mitochondrial dysfunction (Houtkooper et al., 2010). Collectively, there is a tight-knit network of interacting nutrient sensing pathways that govern the cellular metabolic state, in part through modulating mitochondrial function, and thereby influence aging.

### Cellular Senescence and Mitochondrial Dysfunction

Cellular senescence is the transition to quiescence where cells cease dividing, and is characterized by the secretion of inflammatory signaling factors. This can be triggered by different mechanisms such as genomic instability or telomere attrition (McHugh and Gil, 2018). Senescent cells accumulate during aging in all tissues (Hudgins et al., 2018). This accumulation has an effect on cellular homeostasis. It increases inflammation, decreases tissue function and causes stem cell exhaustion, which all contribute to aging (López-Otín et al., 2013; McHugh and Gil, 2018).

One factor that can induce cellular senescence is oxidative stress. Indeed, when complex I, II and III are inhibited, by specific complex inhibitors, this will induce senescence (Yoon et al., 2003; Moiseeva et al., 2009). Similarly, when cells are treated with FCCP to depolarize the mitochondrial membrane potential, senescence is induced (Stöckl et al., 2006). All these findings indicate that defective OXPHOS leads to cellular senescence. Mitochondrial dynamics also play a role in cellular senescence. By knocking out a protein important for fission in mammalian cells, Fis1, mitochondria were elongated, which was accompanied by lower OXPHOS and higher ROS production, and this led to a significantly higher level of senescence (Lee et al., 2007). Another key aspect of senescence is the senescence-associated secretory phenotype (SASP), secreting pro-inflammatory cytokines, proteases and growth factors (Wiley et al., 2016). This secretory phenotype is activated upon mitochondrial dysfunction and depends on AMPK activation (Correia-Melo et al., 2016; Wiley et al., 2016). AMPK will phosphorylate the tumor suppressor gene p53 and this induces senescence (Wiley et al., 2016). These senescent cells will release the SASP, which amplifies the progression of senescence by inducing senescence in neighboring cells (Correia-Melo et al., 2016).

Together these results indicate that defects in OXPHOS, fission/fusion, high ROS production and altered mitochondrial biogenesis induce senescent cells and therefore the aging phenotype.

## Integrating Mitochondrial Function With the Integrative Hallmarks of Aging

Integrative hallmarks of aging are hallmarks that have a more direct effect on the tissue homeostasis and function.

### Stem Cell Exhaustion and Mitochondrial Dysfunction

Stem cells can renew themselves and specific stem cells can give rise to a specific kind of tissue. However, aging reduces the renewal capability of stem cells (Ermolaeva et al., 2018). During aging, telomeres shorten and mutations occur in stem cells, which give rise to senescence of these stem cells. This stem cell exhaustion can, for instance, lead to neurodegenerative diseases or a decline in hematopoiesis which in turn leads to less production of adaptive immune responses (López-Otín et al., 2013; Ermolaeva et al., 2018). Though still a long way from application, it may eventually be possible to reprogram aged somatic stem cells into pluripotent stem cells as a way to prevent or reverse aspects of aging (Ahlqvist et al., 2015).

Stem cell exhaustion can also be caused by mitochondrial dysfunction. Increased ROS production in stem cells reduces the renewal of the stem cells in mice, which was rescued by antioxidant treatment (Jang and Sharkis, 2007). In line with that observation, mutator mice, which express proofreading defects in mitochondrial DNA polymerase gamma and thereby accumulate random mutations in mtDNA, present with an accelerated aging phenotype, demonstrated by graying fur, significant weight loss, osteoporosis and less ATP production in the heart when compared to wild type mice of the same age (Trifunovic et al., 2004). Moreover, these mice had both neural and hematopoietic stem cell dysfunction during their development. The neural stem cells in the mutator mice had decreased self-renewal capacity that was rescued by treating the cells with a ROS inhibitor (Ahlqvist et al., 2012), demonstrating that rescuing the mitochondrial dysfunction could rescue the stem cell dysfunction. Furthermore, lowering the mitochondrial activity in hematopoietic stem cell in mice by using carbonyl cyanide-p-trifluoromethoxyphenylhydrazone (FCCP), which chemically uncouples the electron transport chain, resulted in rapid stem cell differentiation (Vannini et al., 2016). These data suggest that mtDNA maintenance is important for healthy stem cells renewal.

Thus, mitochondrial dysfunction can cause stem cell exhaustion via several mechanisms. At the same time, this creates avenues for intervention since methods to improve mitochondrial function might improve stem cell renewal capacity and hence prevent its contribution to aging.

### Altered Intercellular Communication and Mitochondrial Dysfunction

One example of altered intercellular communication is “inflamm-aging,” which is the development of chronic inflammation in aged people (Ferrucci and Fabbri, 2018). This chronic inflammation includes high levels of pro-inflammatory cytokines such as IL-1, IL-6, IFNα, transforming growth factor beta (TGFβ), and tumor necrosis factor (TNF) secreted by T and B cells (Ferrucci and Fabbri, 2018). Inflamm-aging is a risk factor for cancer, dementia and poor health status (Borrello et al., 2005; Gorelick, 2010; Leonardi et al., 2018). For instance, in many it was shown that the *RET/PTC* oncogene, a thyroid tumor, induces genes which are involved in inflammation, such as chemokines and cytokines (Borrello et al., 2005).

Mitochondria play an important role in altered intercellular communication. Upon inflammation, cells secrete signals to trigger an immune response. Damage associated molecular patterns (DAMPs) are danger signals released by cells if there is stress, apoptosis or necrosis. Mitochondria also secrete DAMPs, including molecules such as ATP, mtDNA and ROS (McGuire, 2019). For instance, it was reported that there is a high increase in mtDNA levels in plasma of people of 50+ years old with concomitant high levels of inflammatory cytokines (Pinti et al., 2014). Mitochondria can also trigger inflammatory responses via the mitochondrial antiviral signaling proteins (MAVS) (Koshiba et al., 2011). These proteins localize on the outer mitochondrial membrane, and phosphorylate interferon-regulatory factors 1/5/7 (IRF1/5/7) in an OXPHOS-dependent manner (Koshiba et al., 2011; Lazear et al., 2013). IRFs activate antiviral responses and this will lead to the production of type I interferon (IFN). Aged monocytes, however, have mitochondrial dysfunction including lower OXPHOS, but also lower IFR3/7. This results in lower IFN synthesis, and lower anti-viral response (Molony et al., 2017; Pence and Yarbro, 2018).

Alongside increased inflammation, the adaptive immune response declines with age. T-cells are important for the adaptive immune response to prevent infections (McGuire, 2019), but in the elderly the T-cell activity is reduced, making them more susceptible for diseases (Pieren et al., 2019). Interestingly, mice with T cells that were specifically deficient in a mitochondrial DNA–stabilizing protein exhibited multiple features associated with aging, including neurological, metabolic, muscular, and cardiovascular impairments (Desdín-Micó et al., 2020). The defective T cells initiated an early inflammatory program that induced premature senescence (Desdín-Micó et al., 2020). Another study showed that T cells from older subjects also had defects in autophagy and mitochondrial bioenergetics when compared to those of younger subjects, and that metformin alleviate aging-associated inflammation enhances autophagy and normalizes mitochondrial function to alleviate aging-associated inflammation (Bharath et al., 2020). T-cells with a cytochrome C oxidase 10 (*Cox10*), which is a part of OXPHOS complex IV, were infected with influenza (Tarasenko et al., 2017). These T-cells were not activated and were immunodeficient (Tarasenko et al., 2017), demonstrating that OXPHOS is important for T-cell activation.

To conclude, altered intercellular communication can cause mitochondrial dysfunction and vice versa, which contributes to age-related inflammation.

## Integrating the Hallmarks of Aging Across the Tree of Life

**FIGURE 1:**
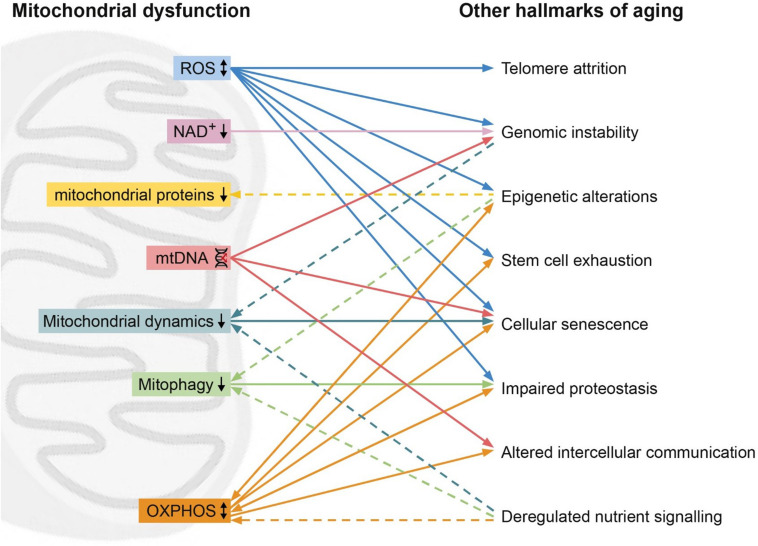
Mitochondrial dysfunction (left) and its relation with other hallmarks (right). High levels of reactive oxygen species (ROS, blue) can lead to telomere attrition, genomic instability, epigenetic alternations, stem cell exhaustion, cellular senescence. On the other hand, ROS can also improve proteostasis. Low levels of NAD^+^ (pink) can lead to genomic instability. Epigenetic alterations can lead to reduced mitochondrial proteins (yellow) such as STEAP2, RHOT2, and RAB32. Mitochondrial DNA (mtDNA) damage (red) can result in genomic instability, cellular senescence and altered intercellular communication. Hallmarks of aging genomic instability and deregulated nutrient sensing can lead to reduced mitochondrial dynamics (turquoise), while reduced mitochondrial dynamics induces cellular senescence. Reduced mitophagy (green) leads to impaired proteostasis, while epigenetic alterations and deregulated nutrient sensing induce reduced mitophagy. Oxidative phosphorylation (OXPHOS, orange) affects the hallmarks epigenetic alterations, stem cell exhaustion, cellular senescence, impaired proteostasis, altered intercellular communication and is in turn affected by deregulated nutrient sensing, epigenetic alterations and impaired proteostasis. Dashed line represents an effect of a hallmarks of aging on the mitochondrial dysfunction.

Since their classification, the nine hallmarks of aging have been used to describe and clarify the cellular and molecular processes involved in aging as a phenotype. However, these hallmarks are often thought of as discrete processes, as opposed to an intertwined system. In this review, we highlight the interrelation between the hallmarks of aging, with a focus on the mitochondria. Unifying mitochondrial factors such as decreased OXPHOS, increased ROS, reduced mitophagy, dysregulated fission or fusion, and mitochondrial proteostasis connect all of the hallmarks (Figure 1).

The majority of mechanistic aging research occurs in model organisms such as *C. elegans*, mice and *D. melanogaster*, and the interactions we describe are based on such traditional experimental models. However, resources such as the AnAge database demonstrate the dramatic variation in organism aging and longevity, which all can contribute to our understanding of these processes (Tacutu et al., 2018). The emergence of new model organisms provides further insight into aging mechanisms. The killifish (*Nothobranchius furzeri*) is a vertebrate, allowing for confirmation of findings in invertebrate organisms before moving to experiments in mammals. For example, the manipulation of the mitonuclear balance in assembly of the respiratory chain was shown to extend lifespan in *C. elegans* (Houtkooper et al., 2013). Recently, using comparative analysis of the genomes of various species within the killifish family, these genes were linked to evolution of lifespans in vertebrates as well (Sahm et al., 2017). These findings demonstrate not only the benefit of studying a wide variety of model organisms, but that comparative studies within the tree of life can also provide important insights into the mechanisms of aging.

However, the same reasons these organisms are often chosen as models can also make them less than suitable representations of the entirety of the phylogenetic tree. For instance, killifish, along with yeast, worms, flies, and mice, are all short-lived and fast-aging (Cohen, 2018). Extremely long-lived species, such as the naked mole rat and turtles, or seemingly immortal species, such as Hydra, can also provide valuable insights into molecular aging, such as the role of mitochondrial dysfunction plays.

The naked mole rat (*Heterocephalus glaber*) is well-known in the aging field as it boasts the most extreme longevity relative to its body size (Gorbunova et al., 2008). This extraordinary lifespan can potentially be connected to mitochondrial function. Naked mole rats exhibit remarkably less age-related changes in mitochondrial mass and efficiency, as well as lipid peroxidation (Buffenstein, 2008; Stoll et al., 2016). Particularly, OXPHOS complex IV expression and activity remains stable throughout lifespan (Stoll et al., 2016). When comparing the skeletal and heart muscle of naked mole rats to mice, one study found that the two species generated essentially equal quantities of mitochondrial H_2_O_2_ as a proxy of overall ROS metabolism. However, naked mole rats had a significantly greater capacity to consume ROS compared to mice (Munro et al., 2019). The naked mole rat therefore demonstrates a clear model for the aging benefits of maintaining mitochondrial function. Another example is birds. Birds have a relatively long lifespan considering their high metabolic activity and small body size, and it is shown that birds have less ROS production and longer telomeres (Hickey et al., 2012). Similarly, it has been noted that hypoxia in the freshwater turtle *Trachemys scripta elegans* inhibits mitochondrial respiration and ROS production, preventing oxidative damage (Bundgaard et al., 2019). These examples illustrate that different species have different ways to maintain mitochondrial function in the face of physiological challenges, and thereby sustain a relatively long and healthy lifespan.

The cnidarian Hydra (*hydra vulgaris*) is capable of continuous self-renewal and is therefore considered immortal (Schaible et al., 2015). This renewal is mainly due to the robust activity of three stem cell populations (Tomczyk et al., 2015). However, Hydra also have a well-described stress response system, including antioxidant processes (Dash et al., 2007). The FoxO transcription factor is expressed in stem cells, and reduction in FoxO levels negatively affect the proliferation of stem cells (Boehm et al., 2012). While there are few mitochondria-specific studies in Hydra, the interaction of these stem cells and FoxO regulation provide an example of interacting hallmarks in regulation of Hydra immortality. Therefore, interactions between hallmarks are not only present outside the classical aging model organisms, but also that all hallmarks connect together, not just within the lens of mitochondrial dysfunction.

## Conclusion

Across the phylogenetic tree, the hallmarks of aging connect to and influence one another. Although we chose to focus our review on the hallmark of mitochondrial dysfunction, a clear pattern emerges that all hallmarks of aging influence one another. For instance, cellular senescence can be induced by genomic instability or telomere attrition (Lidzbarsky et al., 2018) and epigenetic alternations can lead to genomic instability (Pal and Tyler, 2016). It is hence evident that the hallmarks of aging are not discrete entities as how are often presented, but instead operate in a large and tightly connected network. Targeting one factor of this network can result in affecting other hallmarks and thus influence the whole network of aging. Although this complicates our interpretation of anti-aging interventions and requires a more holistic approach, it also opens opportunities for treatment options that not only target one hallmark but in fact act on the entire, or at least a large section of the network. In relation to the phylogenetic tree of life, while the exact details of the hallmarks of aging may differ, the main commonality that unifies aging across all species is the fact that all their hallmarks interconnect. Taking the entirety of this network into account will benefit the aging research community, and ultimately allow for a greater understanding of the aging processes and the progression of age-related disease.

## Author Contributions

All authors listed have made a substantial, direct and intellectual contribution to the work, and approved it for publication.

## Conflict of Interest

The authors declare that the research was conducted in the absence of any commercial or financial relationships that could be construed as a potential conflict of interest.
